# Trace elements in native and improved paddy rice from different climatic regions of Sri Lanka: implications for public health

**DOI:** 10.1186/s40064-016-3547-9

**Published:** 2016-10-24

**Authors:** Saranga Diyabalanage, Thamara Navarathna, Hemalika T. K. Abeysundara, Sanath Rajapakse, Rohana Chandrajith

**Affiliations:** 1 Department of Geology, Faculty of Science, University of Peradeniya, Peradeniya, Sri Lanka; 2 Postgraduate Institute of Science, University of Peradeniya, Peradeniya, Sri Lanka; 3 Bandaranaike Memorial Ayurvedic Research Institute, Navinna, Maharagama, Sri Lanka; 4 Department of Statistics and Computer Science, Faculty of Science, University of Peradeniya, Peradeniya, Sri Lanka; 5 Department of Molecular Biology and Biotechnology, Faculty of Science, University of Peradeniya, Peradeniya, Sri Lanka

**Keywords:** Paddy soils, Indigenous rice, ICP-MS, Selenium deficiency, Chronic kidney disease of uncertain aetiology (CKDu)

## Abstract

**Background:**

Samples of 226 new improved and 21 indigenous rice (*Oryza sativa* L.) varieties were collected from the rice fields in three climatic zones of Sri Lanka and concentrations of 18 trace elements (Li, B, Al, Cr, Mn, Fe, Co, Ni, Cu, Zn, As, Se, Sr, Mo, Cd, Ba, Pb and Bi) were measured giving particular emphasis on Se, Cd and As using ICP-MS. The two way multivariate analysis of variance (MANOVA) method was employed to identify the differences in composition among rice from different climatic zones.

**Results:**

The mean values obtained for both white and red rice were Se (36; 25 µg/kg), As (42; 45 µg/kg) and Cd (70; 123 µg/kg) on dry weight basis. However mean content of Se, As and Cd of native rice varieties were 69, 74 and 33 µg/kg, respectively. Statistical interpretations showed that in the majority of cases, there was a significant difference in Cd content among climatic zones whereas Se and Pb show differences between white and red rice varieties. Arsenic did not indicate any significant difference either between rice types or among climatic regions. Notably Se and As contents in indigenous rice were higher than that of improved rice types. To assess the safety of dietary of intake, daily intake of Se, Cd and As by rice were calculated. Non-gender specific Estimated Daily Intake (EDI) of Se, Cd and As consuming improved rice are 9.31, 24.1 and 12.2 µg day^−1^, respectively.

**Conclusions:**

Since over 50 % of daily meals of people contain rice or rice based products, Se intake is expected to be deficient among the Sri Lankan population.

## Background

Paddy rice (*Oryza sativa* L.) is the second most important staple food for over half of the world’s population and also cultivated throughout all countries (Muthayya et al. 2014). 30 % of the dietary energy supply and 20 % dietary protein intake in Asia are provided by rice (WHO/FAO 2002). However, it is well-known that rice invariably contains significant amounts of trace elements that accumulate through the environmental food chain (Watanabe et al. 1996). Although trace elements in rice are found usually in low quantities, due to application of agrochemicals and irrigation of paddy fields with contaminated water, contents could considerably elevated. For instance, significantly higher amounts of Cd, Pb and As were reported in rice grains from contaminated areas and even from some non-polluted regions (Meharg et al. 2009, 2013; Watanabe et al. 1996). Exposure to such toxic trace elements through food chains could cause an adverse impact on human health leading to certain chronic diseases. Hence serious concerns over heavy metal accumulation in the soil-rice plant system have been addressed in recent years (Gupta and Gupta 1998; Fu et al. 2008; Zhu et al. 2008). In certain regions of Asia, elevated levels of toxic trace elements such as Cd and As in rice were reported. For instance elevated Cd in rice were reported in Bangladesh and Sri Lanka (Meharg et al. 2013) and also in few other Asian countries (Watanabe et al. 1996) while arsenic contaminated rice was reported in Bangladesh (Das et al. 2004; Abedin et al. 2002; Meharg and Rahman 2003).

Trace elements such as Se, Mo, Cr, Mn, Fe, Co, Cu, Zn are well-known as micronutrients that help in the proper functioning of human biological systems, while non-essential elements such as Pb, As, Cd, Hg do not have any clear physiological functions (Underwood 1979; Dissanayake and Chandrajith 1999). From among essential elements, Se plays a great role in human organisms by contributing large number of biological functions mainly enzymatic roles as an antioxidant and as a catalyst for thyroid hormone production. (Rayman 2000; Beckett and Arthur 2005; Sun et al. 2014). Selenium also plays a dual role as an essential micro-nutrient and as a toxic element with a narrow range between the edges of the supply spectrum (Fordyce et al. 2000; Tan et al. 2002). In Asia, most of the human Se requirement is supplied via cereals such as rice but supplementation is considerably low causing serious health problems (Williams et al. 2009). Therefore assessing Se levels in rice is extremely important since the majority of Se requirement is supplied from rice.

**Fig. 1:**
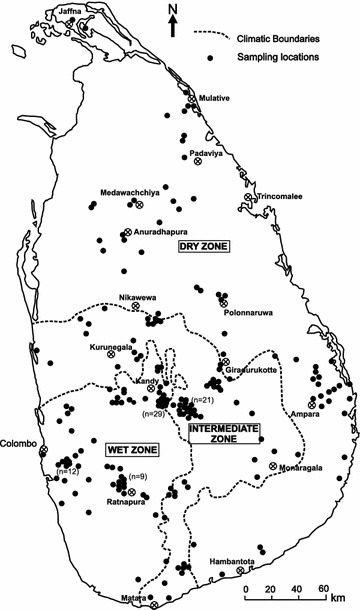
Sampling locations of rice from Sri Lanka with respect to climatic zones

Similar to other counties in Asia, rice is the staple food in Sri Lanka and is deeply embedded with its economy, traditions and culture. Rice is grown in the entire island which is characterized with variable climate and geography. It is estimated that nearly 34 % (0.77 million ha) of the total cultivated areas in Sri Lanka have been used for rice cultivations from which over 95 % of the domestic requirement is fulfilled (DOASL 2015). Both rain-fed and irrigated rice paddies are common in Sri Lanka which is characterized by three distinct climatic zones known as the wet, intermediate and dry zones (Fig. 1). The wet zone receives over 2500 mm annual rainfall while the dry zone receives about 1000 mm of annual rain. The intermediate zone with about 1500 mm annual rain is located in between wet and dry zones (Domrös 1979). Monsoonal rainfalls from north-east (NE) and south-west (SW) of the island control the spatial variability of the climate of Sri Lanka. The wet zone rice cultivation is mainly rain fed, but due to scarcity of water, cascading system of reservoirs and canal irrigations (Panabokke et al. 2002; Mahatantila et al. 2008) are used for the paddy cultivation in the intermediate and dry zone regions.

In recent years, wide attention was paid to the quality of drinking water and food in Sri Lanka due to the emergence of geographically distributed health issues. As a tropical island, Sri Lanka is strongly interlinked between geographically distributed endemic health issues and environmental factors (Dissanayake and Chandrajith 2007, 1999). Among such health incidences, Chronic Kidney Disease of uncertain aetiology (CKDu) that is prevalent in the dry zone areas of Sri Lanka has received wide attention (Athuraliya et al. 2011; Chandrajith et al. 2011). The histopathological investigations on CKDu suggested an involvement of a possible environmental nephrotoxin (Athuraliya et al. 2011; Nanayakkara et al. 2012). Recent studies indicated high levels of toxic elements such as Cd (Meharg et al. 2013) and As (Jayasumana et al. 2013) in rice that was attributed to the aetiology of endemic CKDu in Sri Lanka. Jayatilake et al. (2013) inferred a possible link between deficiency of selenium and incidence of CKDu. Rice grains can be contaminated with toxic elements resulting from agrogenic processes such as extensive use of fertilizers and pesticides that are common in contemporary rice field agriculture in Sri Lanka. In tropical Sri Lanka, different new improved varieties of genotype Indica rice are widely cultivated and are more popular among farmers due to more economic benefits with higher yield, drought and flood tolerance and disease resistance. It was reported that over 2000 rice varieties were cultivated in Sri Lanka, but only a few are now widespread (Paroda 1999; Kennedy and Burlingame 2003). At present, several white and red types of improved rice types are widely cultivated in Sri Lanka (DOASL 2015). About 16 native varieties of rice are also grown in isolated paddy fields by mostly adapting organic agriculture practices. Therefore, the main objective of this study was to assess the levels of toxic trace elements in rice produced in Sri Lanka giving particular emphasis to Se, As and Cd since these elements were considered as important causative agents of CKDu. The toxic trace element levels were compared between different rice types (white and red) and their geographic locations in respect to the climatic zones.

## Methods

A total of 226 samples of new improved rice and 21 samples of native rice varieties were collected, covering most parts of the rice growing regions in Sri Lanka (Fig. 1). From new improved rice samples, 163 samples were white rice and 81 samples from wet zone, 70 from intermediate zone and 75 from dry zone were collected. However almost all traditional varieties were obtained from wet zone paddies. Samples of rice seeds were directly collected from paddy fields and stored in zip-lock bags. Seeds were then dried at 40 °C to remove moisture and de-husked to separate grains that were finely powdered using an agate mortar and pestle. Samples were dried for a few hours at 60 °C and were stored at 4 °C in screw-capped plastic containers. About 0.50 g of the subsamples were digested using Mars-6 microwave digester (CEM; Matthews, NC) equipped with EasyPrep Plus high pressure vessels. Digestion was performed with 10 mL HNO_3_ (≥69.0 % TraceSelect; Fluka, Switzerland) and 1 mL of H_2_O_2_ (35 wt. %; Sigma-Aldrich, Germany) and diluted to 50 ml using ultra-pure water. Concentration of trace elements Li, B, Al, Cr, Mn, Fe, Co, Ni, Cu, Zn, As, Se, Sr, Mo, Cd, Ba, Pb and Bi were determined using Thermo ICapQ (Thermo-Fisher Scientific Inc., Bremen, Germany) Inductively Coupled Plasma Mass Spectrometry (ICP-MS). Multi-element ICP-MS standards (Sigma-Aldrich, Germany) were used for the instrumental calibration and ^103^Rh was used as the internal calibration standard. All measurements were done at least in duplicates and analytical results were expressed on dry matter basis. The NIST 1568a rice flour reference (National Institute of Standard Technology; MD, USA) material was used to validate the analytical procedure. We have obtained 0.330 ± 0.004 mg/kg for Se against 0.38 ± 0.04 mg/kg; 0.260 ± 0.003 mg/kg for As against 0.29 ± 0.03 mg/kg and 0.0280 ± 0.003 mg/kg for Cd against 0.022 ± 0.002 mg/kg with NIST 1568a. For Cu, Zn and Mn recoveries were within ±10 % while slightly higher recoveries (±15 % RSD) were observed for Mn and Al with the NIST 1568a.

All statistical analyses were performed by SPSS ver. 16.0 in which concentrations less than the limit of detection (LOD) are allocated a value of half-LOD for subsequent statistical interpretations. Two way multivariate analysis of variance (MANOVA) was applied to evaluate the differences in elemental composition in rice between climatic zones and the rice type (white or red). In this case, the overall mean of the groups were compared by test statistics. In all cases probability value <0.05 (95 % level) was considered as significant. Non-gender specific Estimated Daily Intake (EDI) of trace elements through rice consumption were estimated as EDI = C_*i*_ × Q, where C_*i*_ is the average concentration of element *i* and Q is the amount of average daily consumption of rice which is considered as 284 g/day (Fordyce et al. 2000).

## Results and discussion

In Sri Lanka, either white or red improved rice types are widely cultivated. Application of chemical fertilizers and pesticides is common practice in rice cultivations anticipating higher economic returns. Despite the high nutritional value, native varieties are cultivated in isolation mainly using organic agriculture practices. Most of the native varieties contain higher level of glutamic acid, vitamins, and low glycemic index (Gunaratne et al. 2013). The descriptive statistics of trace elements determined in collected rice varieties are shown in Table 1. Although the 18 trace elements were measured using ICP-MS, particular attention was given to elements Se, Cd and As that are discussed in a wider context compared to others.

**Table 1:** Summary statistics of trace elements in rice grains from different climatic zone of Sri Lanka (in µg/kg in dry weight basis; SD-standard deviation)

	Li	B	Al	Cr	Mn	Fe	Co	Ni	Cu	Zn	As	Se	Sr	Mo	Cd	Ba	Pb	Bi
*Wet zone (n* = *81)*																		
Min	2.5	2.5	2987	36.8	2995	3481	1.8	74.2	510	4525	2.48	2.32	31.2	144	0.6	231	2.47	0.32
Max	61	1931	23,976	1113	34,716	46,745	429	7641	6770	33,745	213	168	859	2108	576	4006	1277	86.6
Mean	10	839	8183	277	18,086	14,078	77	1026	2467	15,473	48	30	319	787	128	1433	257	4.74
Median	2	777	6876	203	18,038	11,833	44	571	2333	15,522	40	19	281	745	79	1243	192	2
SD	11	427	3700	215	8235	8234	92	1342	1148	4994	40.8	33.8	193	401	137	824	286	12
*Intermediate zone (n* = *70)*																		
Min	2.5	2.5	2744	13	1798	2036	1.2	65	442	2532	2.5	0.54	14.6	0.88	2.34	176	2.48	2.47
Max	37	1543	16,972	520	28,448	40,589	296	6854	4802	21,320	143	156	720	1953	213	3060	945	69
Mean	6.5	650	8145	219	13,419	12,079	81	1012	2264	11,672	38	24	213	762	54	988	271	4
Median	2.5	611	7865	230	14,006	10,811	67	466	2212	11,669	34	14	184	712	38	898	254	2.5
SD	7	356	2802	129	7317	7806	66	1436	1080	5086	31	29	158	389	45	563	227	8
*Dry zone (n* = *75)*																		
Min	2.5	2.5	3273	23	2139	1724	6.5	51	381	2772	2.5	2.1	28.5	3.0	0.6	209.7	0.6	2.5
Max	61.4	4622	13,700	362	41,710	41,614	398	2518	5547	33,247	186	261	1076	1857	282	3631	916	14.4
Mean	8.5	1016	7705	154	16,153	13,666	82	577	2430	13,156	41	44	342	673	68	1152	322	3.3
Median	2	859	7537	156	17,627	12,280	63	366	2156	13,950	29	26	288	597	52	990	242	2
SD	10.8	915	2534	85	11,272	9377	80	581	1267	6947	39	55	243	446	66	731	239	2.2
*Native rice (n* = *21)*																		
Min	2.5	75.3	3441	160	10,354	5838	21	148	595	7603	2.5	2.5	102	300	8.0	532	1.5	2.5
Max	40	1962	39,789	614	39,467	44,135	199	2262	3488	34,780	143	192	1142	1055	72	3173	1338	39
Mean	9	1137	10,353	285	20,729	20,136	103	542	2167	22,605	74	69	480	573	33	1622	385	11
Median	2	1154	7809	241	19,750	19,529	95	394	2074	21,410	80	78	394	524	27	1647	110	7
SD	11	498	8702	109	6993	9006	58	516	833	5597	42	64	281	200	19	720	536	11
*Sri Lanka improved (all; n* = *226)*																		
Min	2.5	2.5	2744	13.3	1798	1724	1.2	50.9	381	2532	2.5	0.5	14.6	0.9	0.6	176	0.6	0.3
Max	61	4622	23,976	1113	41,710	46,745	429	7641	6770	33,745	213	261	1076	2108	576	4006	1277	87
Mean	8.3	839	8013	218	16,010	13,319	80	876	2392	13,527	43	33	294	741	85	1203	283	4.1
Median	2.5	750	7434	182	16,809	11,696	57	453	2212	14,149	34	19	257	681	55	1083	236	2.5
SD	10	634	3071	163	9278	8512	80	1198	1167	5934	38	42	208	414	99	742	254	8
*Improved white rice (n* = *163)*																		
Min	2.5	2.5	2744	13.3	1798	1724	6.5	51	558	2532	2.5	0.5	19.7	0.9	0.6	232	0.6	0.3
Max	61	4622	38,161	1002	41,710	46,745	429	7641	5276	33,247	186	261	1076	2108	344	4006	1277	69
Mean	8.3	868	8250	210	16,453	13,117	95	944	2434	13,526	42	36	304	741	70	1245	314	3.5
Median	2.5	772	7535	180	17,464	11,673	68	491	2353	14,162	34	25	259	678	47	1083	263	2.5
SD	10.2	658	4098	142	9456	8097	89	1262	1101	5740	34	44	212	418	66	749	263	5.8
*Improved red rice (n* = *63)*																		
Min	2.5	2.5	4766	23	2476	2936	1.2	56	381	3205	2.5	2.1	15	3.0	2.5	176	2.5	1.2
Max	37	3136	14,031	1113	34,716	43,346	128	5087	6770	33,745	213	125	766	2037	576	3005	860	87
Mean	8.4	763	7894	240	14,871	13,829	42	693	2283	13,527	45	25	267	742	123	1097	203	5.5
Median	2.5	693	7192	188	15,851	12,118	38	314	2117	14,128	34	8	241	700	73	1078	120	2.5
SD	8.7	561	2267	207	8773	9529	27	995	1324	6452	46	35	195	408	149	717	211	13

### Selenium in rice

Selenium is one of the important micronutrients investigated in this study since rice is the main source of selenium intake in Sri Lankan population that consumes rice almost three times a day and rice flour is also used very commonly in other food recipes. Nutritional functions of selenium are attained by 25 seleno-proteins, many of them are important for human health. Seleno-proteins act as antioxidant and detoxifying agents (e.g.: Glutathione peroxidase and thioredoxin reductase) and also considered as chemo-protective agents (Combs 2004; Davis et al. 2002; Ferri et al. 2007; Sun et al. 2014). Selenium deficiency has been identified as one of the major health problems among one billion people around the world (Haug et al. 2007). Deficiency of selenium has also been recently implicated to the CKDu in Sri Lanka (Jayatilake et al. 2013). The fact that the recommended average selenium intake varies according to the geographic region, 60 µg per day for men and 53 µg per day for women is generally recommended (Rayman 2000), and over 400 µg per day is considered to be toxic (WHO 1996). Sri Lanka is recognized as a terrain where the Se is deficient in the environment and this fact is attributed to the prevalence of goiter in the wet zone (Fordyce et al. 2000) and CKDu in the dry zone (Jayatilake et al. 2013).

**Fig. 2:**
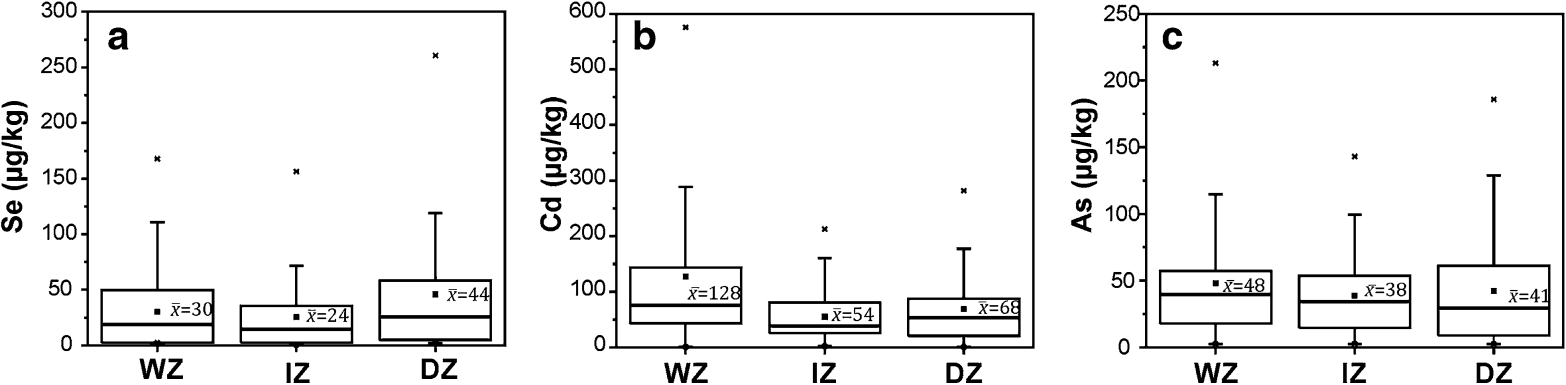
*Box* and *whisker plot* showing (**a**) Se, (**b**) Cd and (**c**) As contents in wet zone (WZ), intermediate zone (IZ) and dry zone (DZ) of Sri Lanka

**Fig. 3:**
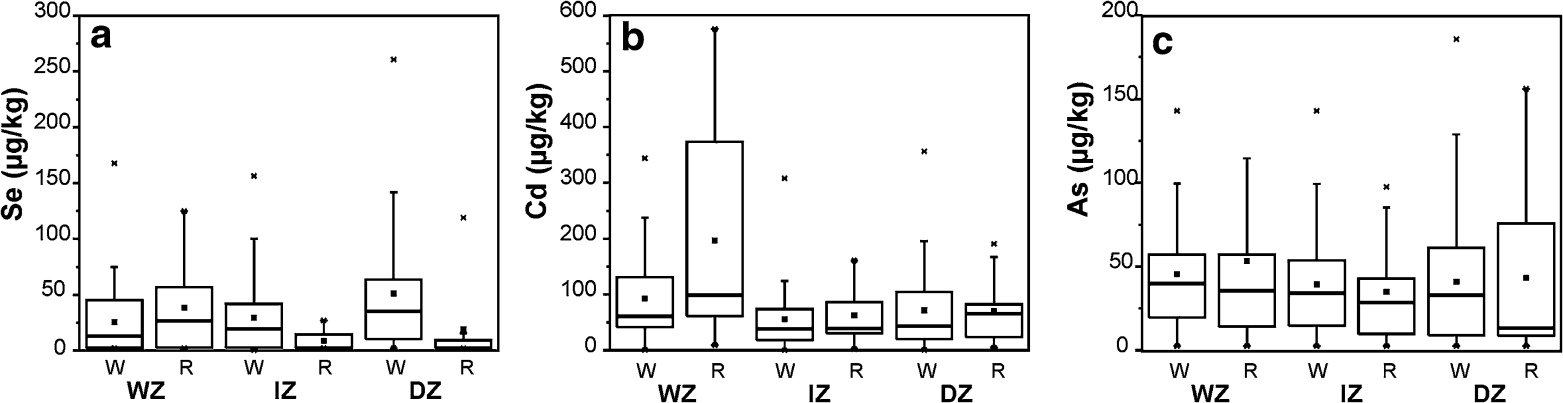
*Box* and *whisker plot* showing (**a**) Se, (**b**) Cd and (**c**) As contents in white (W) and red (R) improved rice types from wet zone (WZ), intermediate zone (IZ) and dry zone (DZ) of Sri Lanka

**Fig. 4:**
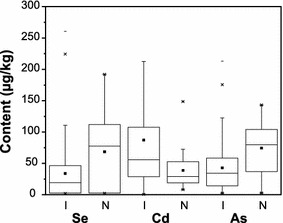
*Box* and *whisker plot* showing Se, Cd and As contents in improved (I) and native (N) rice types of Sri Lanka

**Fig. 5:**
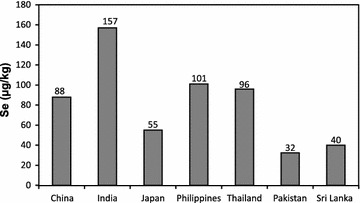
Se content in rice from some Asian counties

Selenium content in rice is determined by the geochemical properties such as mineralogy of the parent material, soil processes (pH, redox state, water logging and submerging conditions) and also by the genotype of the plant (Cao et al. 2001). Physiological importance of Se to higher plants is not yet proven (Lyons et al. 2005) hence, the deficiencies in soil Se content will not effect on rice growth or yield. Flooded conditions in the paddy fields create anaerobic conditions which results in low Se availability for plant uptake. Plant root system takes up Se in the forms of selenate (SeO_4_^2−^), selenite (SeO_3_^2−^) and also as organic compounds (White and Broadley 2009) through sulfate transporters in the root plasma membranes (Bitterli et al. 2010). Selenate and Selenite are the predominant forms of Se for plants whereas elemental Se (Se^0^) and metal selenides (Se^2−^) are not available for plant uptake (Abrams et al. 1990). Selenate is favored under alkaline and oxic conditions whereas Selenite is favored by acidic to neutral soils with low oxic conditions (Neal and Sposito 1989; Bitterli et al. 2010). However, the mobility and bioavailability of selenite is limited due to its higher adsorption affinity to clay minerals, organic compounds and hydroxides. Based on the results of this study, the average Se content in Sri Lankan improved rice varieties were 33 µg/kg. However dry zone rice had a higher mean Se content (44 µg/kg; 2.1–261 µg/kg) than the other two climatic regions (Fig. 2a). Fordyce et al. (2000) also showed that Se content in dry zone rice varies from 6.8 to 150 µg/kg (n = 5). The lower availability of Se in paddy soil might be due to reduction of selenite under water logged conditions (Cao et al. 2001) that may leads to lower content of Se in rice from the wet zone as compared to the dry zone. From studied samples, white grain rice showed a higher mean Se content (36 µg/kg) compared to the red type (25 µg/kg) (Fig. 3a). Interestingly native rice varieties showed remarkably higher Se contents (69 µg/kg) compared to improved varieties (Fig. 4) possibly due to the inherent genetic differences. The content of Se in rice from Sri Lanka is lower compared to rice in other Asian countries (Fig. 5). The mean Se in rice from neighboring India was reported to be as high as 141 µg/kg (35–371 µg/kg) (Williams et al. 2009) and 125 µg/kg (5.3–233 µg/kg) (Kelly et al. 2002). However the mean Se content of Sri Lankan improved varieties is closely similar to the rice from Pakistan (mean 32.4 µg/kg Se; n = 5) (Kelly et al. 2002). Selenium content in paddy soils of Sri Lanka is also deficient or marginal (0.113–5.238 mg/kg) compared to soils in other parts of the world, while higher values of Se were reported in wet zone paddy soils (Fordyce et al. 2000). In spite of higher Se in paddy soils of the wet zone, a lower Se content was noted in this zone. We have calculated the average intake of Se for rice or rice based products from the wet zone, dry zone and intermediate zones and are 8.5, 12.5 and 6.8 µg/day, respectively (Table 2). These values are much lower than the recommended average selenium requirements. The intake is significantly higher (20 µg/day) for a person who consumes native rice; however a majority of the people consumes either white or red improved rice due to the non-availability and the higher cost of native rice varieties.

**Table 2:** Daily intake of Se, Cd and As from improved rice types and native rice varieties

Category	Mean (µg/kg)			Daily intake (µg)			Daily intake (µg per kg body weight)		
	Se	Cd	As	Se	Cd	As	Se	Cd	As
Wet zone	30	128	48	8.5	36.3	13.9	0.14	0.60	0.23
Intermediate zone	24	54	38	6.8	15.3	10.9	0.11	0.25	0.18
Dry zone	44	68	41	12.5	19.2	11.8	0.21	0.32	0.20
Native rice	69	33	74	19.5	9.47	21.2	0.32	0.16	0.35
Sri Lanka	33	85	43	9.31	24.1	12.2	0.16	0.40	0.20

Daily rice consumption (kg) 0.284 (after Fordyce et al. 2000)

Average body weight of adult: 60 kg

### Cadmium in rice

Cadmium is a well-known carcinogen to humans and can also cause anemia, hypertension and severe damage to kidneys, lungs and bones (Khaniki and Zozali 2005). High Cd concentrations in rice are a widespread problem in many parts of the world and the situation is severe in South Asian countries (Meharg et al. 2013). It has also been identified that the bran of rice contains higher amounts of Cd than polished rice (Zhang et al. 1998). The extensive use of phosphate fertilizer for paddy cultivation and irrigation of contaminated water are the main sources of Cd in rice grains. As noted by Watanabe et al. (1996), high average Cd content was recorded from Japan (55.7 µg/kg; n = 788) whereas lowest recorded from Australia (2.67 µg/kg; n = 8). Sri Lanka has also been identified as one of the countries where the dietary intake of cadmium is known to be high (Bandara et al. 2008). Meharg et al. (2013) reported 81 µg/kg of Cd (n = 75), with median of 24 µg/kg (Minimum <0.5 µg/kg and Maximum 800 µg/kg) in rice from Sri Lanka. It is only second to Bangladesh (mean = 99 µg/kg; n = 260). In another study, the Cd in rice was reported to be 1.7–92.5 µg/kg with a mean of 23.36 µg/kg (Bandara et al. 2008).

Based on this study, the average Cd in improved rice varieties were 85 µg/kg (median 55 µg/kg) whereas improved rice grains from wet zone had 128 µg/kg of Cd that is significantly higher compared to rice from the intermediate zone (54 µg/kg) and the dry zone (68 µg/kg) (Fig. 2b). Mobilization of Cd in soil solution is sensitive to redox conditions in which mobilization is more favored under reducing conditions (Chuan et al. 1996). The heavy rainfall associated with the wet zone regions results in prolonged submerging of paddy soils resulting in extreme reducing conditions (Chandrajith et al. 2005b). This facilitates the mobilization of Cd and the subsequent uptake of this by the rice plant. Dry zone paddy soils are irrigated at rates that prevent prolonged submergence that may facilitate binding of Cd into oxides and/or oxyhydroxides of Fe and Mn (Chuan et al. 1996). Phosphate fertilizers particularly superphosphates are the major host of Cd in rice paddy fields and from 2.3 to 46 mg/kg of Cd were reported in triple superphosphates in Sri Lanka (Chandrajith et al. 2010). Compared to white rice (70 µg/kg), red rice (123 µg/kg) had a significantly higher Cd level (Fig. 3b) but the wet zone red rice showed a skewed distribution of Cd content from 9.6 to 576 µg/kg. Native rice varieties of Sri Lanka showed a mean Cd content of 33 µg/kg (median = 27 µg/kg) that is significantly lower than the improved varieties (Fig. 4). Application of organic fertilizer would be the reason for lower Cd content in native rice samples. The mean Cd content of rice in some neighboring countries are 78 µg/kg (India); 50 µg/kg (Nepal) and 27 µg/kg (Thailand), most of which are much lower than the Cd content in Sri Lankan rice (Meharg et al. 2009). However over 92 % of the improved rice samples collected from Sri Lanka contained Cd at a level less than the Codex Committee on Food Additives and Contaminants (CCFAC) maximum permissible level for rice grain of 200 µg/kg (FAO/WHO 2006). As already mentioned, rice is the staple diet of the Sri Lankan population that accounts for over 50 % of the dry diet that provides about 1000 calories. The average Cd intake from improved rice is 24 µg/day while wet zone rice provide 36 µg/day Cd which is significantly higher compared to intake from the dry zone (19 µg/day) and the intermediate zone (15 µg/day). The intake is lower (9.47 µg/day) for a person who consumes native rice (Table 2).

### Arsenic in rice

Arsenic (As) is a metalloid that can cause adverse health effects. For instance, due to consumption of As contaminated water and food, millions of people in Bangladesh are suffering chronic arsenic poisoning (Das et al. 2004). Rice is considered as one of the significant dietary sources of inorganic arsenic (Abedin et al. 2002; Gilbert et al. 2015). Arsenic concentration in rice grain varies widely depending on the regional environmental factors. Rice can accumulate elevated levels of arsenic compared to other cereals in which approximately 80 % of the total arsenic is in the inorganic form (Williams et al. 2007; Zhu et al. 2008). Higher accumulation of As were reported in Bengal Delta where rice fields are irrigated with arsenic contaminated water (Meharg and Rahman 2003). Williams et al. (2007) reported that the baseline inorganic As levels in rice are tenfold higher compared to other cereals. Although the groundwater and surface water arsenic levels are insignificant in Sri Lanka (Chandrajith et al. 2011; Nanayakkara et al. 2014), some studies highlighted alarming higher levels (21–540 µg/kg) of As in rice from Sri Lanka (Jayasumana et al. 2015). This enriched As in rice was attributed to the application of contaminated fertilizer and pesticides in paddy cultivation. However this study indicated a mean of 43 µg/kg As (2.50–213 µg/kg; median 34 µg/kg) in improved rice from Sri Lanka. These values are significantly lower than the previously reported values. Interestingly no significant variation in mean As contents were observed among three climatic zones (Fig. 2c) and also between white (42 µg/kg) and red (45 µg/kg) types (Fig. 3c). Even paddy soils of wet and dry zone did not show remarkable differences in their mean As contents in which wet zone had 0.9 mg/kg and dry zone paddy soils showed 0.7 mg/kg (Chandrajith et al. 2005a). However native rice showed a higher mean As content (74 µg/kg) compared to improved rice varieties (Fig. 4). Higher accumulation of As in native rice can be possibly attributed to the application of organic fertilizer. Arsenic is a highly redox sensitive element that readily combines with iron hydroxides and/or oxyhydroxides or associated with sulfides. The decomposition of compost fertilizer in flooded paddy fields causes the arsenic to mobilize as arsenite and subsequent plant uptake (Takahashi et al. 2004). But none of the rice samples exceeded the total As level (300 µg/kg) recommended for rice and only one sample from wet zone exceeded the recommended level of inorganic arsenic (200 µg/kg) by the Codex Committee on Food Additives and Contaminants and (FAO/WHO 2012). Furthermore, rice plants taken up Arsenite (As^3+^; as H_3_AsO_3_ and H_2_AsO_3_^−^) and Arsenate (As^+5^; as HAsO_4_^2−^ and H_2_AsO_4_^−^) via Si transportes and phosphate cotransporters respectively (Norton et al. 2010). It has been reported that high Silicic acid and Phosphate conditions in the soil solution may reduce As uptake by the rice plant (Bogdan and Schenk 2009; Li et al. 2009). The calculated average intake of As from wet zone improved rice is estimated to be 14 µg/kg, while it is 11 and 12 µg/kg for rice from intermediate zone and dry zone, respectively (Table 2). Interestingly the consumption of native varieties provide higher As intake (21 µg/kg).

### Other trace elements

**Fig. 6:**
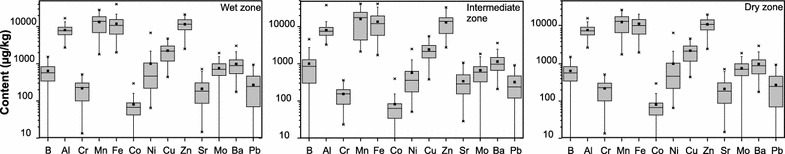
*Box* and *whisker plot* showing trace elements in improved rice from three climatic zones of Sri Lanka

It is very important to understand the accumulation of trace elements in rice grains since some of the trace elements are toxic while some others are considered as essential. Particularly toxic trace elements can cause profound effects on health of people who consume contaminated rice. Summary data of studied trace elements are shown in Table 1. From among all improved rice samples, concentrations of Al (8013 µg/kg), Mn (16,010 µg/kg), Fe (13,319 µg/kg) and Zn (13,527 µg/kg) ranged over four orders of magnitude whereas concentrations of Cu (2392 µg/kg); Ba (1203 µg/kg) and Ni (876 µg/kg) ranged over three orders of magnitude (Fig. 6). Among the other studied trace elements, the mean contents of B (839 µg/kg); Mo (741 µg/kg); Sr (294 µg/kg); Pb (283 µg/kg); Cr (218 µg/kg); Co (80 µg/kg); Bi (4 µg/kg); and Li (8 µg/kg) are comparatively low. From among these elements Fe, Zn, Cu, Mo and B are considered as essential trace elements for the human health (Dissanayake and Chandrajith 1999). Compared to improved rice samples, native rice showed a slightly higher Pb content. In general, Li, Ni, Zn, Mn and Bi are higher in wet zone rice while B is higher in dry zone rice. Usually Cu and Zn in Indica rice are higher than Japonica rice and Zn is higher in Indica white rice (Yang et al. 1998).

### Multivariate analysis of variance (MANOVA)

In order to assess significant differences in total metal concentration or individual elements and group variabilities i.e. climatic zones and rice varieties, multivariate analysis of variance (MANOVA) was used (Table 3). An assumption was made on the significant difference of trace elements in terms of climatic zones (wet, dry and intermediate) and rice types (white and red). In MANOVA, inter-correlations of the independent variables are taken into account in which all variables were considered multivariate (Todorov and Filzmoser 2010) and overall mean of the group is compared by Wilks’ Lambda, Lawley-Hotelling, Pillai’s Trace and Roy’s Largest Root statistics and between group variances is expressed as F-statistics. Only improved rice samples were considered for the MANOVA test. The overall results of the MANOVA for improved rice from Sri Lanka are shown in Table 4. As shown in the results, with respect to all variables (p > 0.01) the overall compositions of rice are not significantly different from among groups such as climatic zones and rice types (Table 3). However when individual elements are considered, independent variables (Table 4) and significant differences were observed in Se, Cd, Cr, Cu, Zn and Ba contents among climatic zones and between rice types. The contents of Mn and Sr only showed a significant difference among climatic zones while Co and Pb only showed significant differences between white and red types. Interestingly As content does not show any significant difference between neither climatic zone nor rice type.

**Table 3:** Results of MANOVA multivariate tests for rice samples of three climatic zones and two rice types (white and red)

Test	Value	*F*	Hypothesis d.f.	Error d.f.	Sig.
*Climatic zones*					
Pillai’s Trace	0.564	3.837	36.000	352.000	0.000
Wilks’ Lambda	0.508	3.916^a^	36.000	350.000	0.000
Hotelling’s Trace	0.826	3.994	36.000	348.000	0.000
Roy’s largest root	0.584	5.713^b^	18.000	176.000	0.000
*Rice type*					
Pillai’s Trace	0.230	2.906^a^	18.000	175.000	0.000
Wilks’ Lambda	0.770	2.906^a^	18.000	175.000	0.000
Hotelling’s Trace	0.299	2.906^a^	18.000	175.000	0.000
Roy’s largest root	0.299	2.906^a^	18.000	175.000	0.000

^a^Exact statistic

^b^The statistic is an upper bound on F that yields a lower bound on the significance level

**Table 4:** Multivariate tests (MANOVA) results for rice samples that considered as climatic zones, rice types and both

Element	Significance level		
	Climatic zones	Rice types	Zone-types
Se	0.214	*0.006*	*0.003*
As	0.075	0.709	0.641
Cd	*0.000*	*0.038*	*0.006*
Li	0.047	0.554	0.127
B	0.132	0.250	0.189
Al	0.912	0.796	0.438
Cr	*0.000*	0.603	*0.009*
Mn	*0.010*	0.116	0.117
Fe	0.547	0.507	0.363
Co	0.917	*0.000*	0.936
Ni	0.050	0.077	0.795
Cu	0.183	*0.009*	*0.020*
Zn	*0.000*	0.621	*0.032*
Sr	*0.003*	0.139	0.053
Mo	0.082	0.773	0.256
Ba	*0.000*	0.090	*0.015*
Pb	0.404	*0.018*	0.971
Bi	0.338	0.254	0.240

Italic values indicate significance of p values; p < 0.05

## Conclusions

Rice is one of the most widely consumed foods worldwide while in Sri Lanka it is consumed at a rate over 100 kg/person rice annually. Recently it has been reported that rice in Sri Lanka contains high levels of highly toxic elements such as cadmium and arsenic. Such elements were then attributed to the onset of chronic kidney diseases in certain geographically discrete areas of the country. The results obtained in this study increase our attention of trace metal contents in rice from different climatic regions and also difference between indigenous rice and improved rice varieties. A multivariate data analysis method was used to identify the difference in trace metal composition in improved rice on climatic condition hence the soil characteristics and on different varieties as white and red rice.

Based on the results of descriptive statistics and multivariate analyses, it can be concluded that the As content in rice does not significantly differ among climatic zones and rice types while Se differs among rice types. However Cd content in rice differs drastically according to rice type and climate zone. The elevated levels of toxic elements, Cd and As in rice suggests that paddy soils have been subject to a high input of anthropogenic contaminants that are most likely related to the agrogenic activities such as application of fertilizer used to improve production and quality since the geogenic input is not considerable in Sri Lanka. Interestingly, native rice varieties contained significantly higher content of Se which is an essential trace element that is considered as being deficient among Sri Lankan population. Since the Se intake among Sri Lankans seems to be low and critical while the main staple food does not supply adequate amounts, assessing the Glutathione peroxidase in blood in the population is highly warranted. It is also important to adopt regulations to control the agrogenic trace metal inputs.

## References

[CR1] Abedin MJ, Cresser MS, Meharg AA, Feldmann J, Cotter-Howells J (2002). Arsenic accumulation and metabolism in rice (*Oryza sativa* L.). Environ Sci Technol.

[CR2] Abrams M, Shennan C, Zasoski R, Burau R (1990). Selenomethionine uptake by wheat seedlings. Agron J.

[CR3] Athuraliya NTC, Abeysekera DTJ, Amerasinghe PH, Kumarasiri R, Bandara P, Karunaratne U, Milton AH, Jones AL (2011). Uncertain etiologies of proteinuric-chronic kidney disease in rural Sri Lanka. Kidney Int.

[CR4] Bandara JMRS, Senevirathna DMAN, Dasanayake DMRSB, Herath V, Bandara JMRP, Abeysekara T, Rajapaksha KH (2008). Chronic renal failure among farm families in cascade irrigation systems in Sri Lanka associated with elevated dietary cadmium levels in rice and freshwater fish (Tilapia). Environ Geochem Health.

[CR5] Beckett GJ, Arthur JR (2005). Selenium and endocrine systems. J Endocrinol.

[CR6] Bitterli C, Bañuelos G, Schulin R (2010). Use of transfer factors to characterize uptake of selenium by plants. J Geochem Explor.

[CR7] Bogdan K, Schenk MK (2009). Evaluation of soil characteristics potentially affecting arsenic concentration in paddy rice (*Oryza sativa* L.). Environ Pollut.

[CR8] Cao ZH, Wang XC, Yao DH, Zhang XL, Wong MH (2001). Selenium geochemistry of paddy soils in Yangtze River Delta. Environ Int.

[CR9] Chandrajith R, Dissanayake CB, Tobschall HJ (2005). The abundances of rarer trace elements in paddy (rice) soils of Sri Lanka. Chemosphere.

[CR10] Chandrajith R, Dissanayake CB, Tobschall HJ (2005). Geochemistry of trace elements in paddy (rice) soils of Sri Lanka–implications for iodine deficiency disorders (IDD). Environ Geochem Health.

[CR11] Chandrajith R, Seneviratna S, Wickramaarachchi K, Attanayake T, Aturaliya TNC, Dissanayake CB (2010). Natural radionuclides and trace elements in rice field soils in relation to fertilizer application: study of a chronic kidney disease area in Sri Lanka. Environ Earth Sci.

[CR12] Chandrajith R, Nanayakkara S, Itai K, Aturaliya TNC, Dissanayake CB, Abeysekera T, Harada K, Watanabe T, Koizumi A (2011). Chronic kidney diseases of uncertain etiology (CKDue) in Sri Lanka: geographic distribution and environmental implications. Environ Geochem Health.

[CR13] Chuan MC, Shu GY, Liu JC (1996). Solubility of heavy metals in a contaminated soil: effects of redox potential and pH. Water Air Soil Pollut.

[CR14] Combs GF (2004). Status of selenium in prostate cancer prevention. Br J Cancer.

[CR15] Das HK, Mitra AK, Sengupta PK, Hossain A, Islam F, Rabbani GH (2004). Arsenic concentrations in rice, vegetables, and fish in Bangladesh: a preliminary study. Environ Int.

[CR16] Davis CD, Zeng H, Finley JW (2002). Selenium-enriched broccoli decreases intestinal tumorigenesis in multiple intestinal neoplasia mice. J Nutr.

[CR17] Dissanayake CB, Chandrajith R (1999). Medical geochemistry of tropical environments. Earth Sci Rev.

[CR18] Dissanayake CB, Chandrajith R (2007). Medical geology in tropical countries with special reference to Sri Lanka. Environ Geochem Health.

[CR19] DOASL (2015) Crop recommendations. Department of agriculture, Govenment of Sri Lanka, http://www.agridept.gov.lk/index.php/en/crop-recommendations/808. http://www.agridept.gov.lk/index.php/en/crop-recommendations/808. Accessed 25 Oct 2015

[CR20] Domrös M (1979). Monsoon and land use in Sri Lanka. GeoJournal.

[CR21] FAO/WHO (2006) Report of the 38th session of the Codex Committee on food additives and contaminants. Joint FAO/WHO food standards programme codex alimentarius commission, 29th Session, Geneva, Switzerland, 3–7 July 2006

[CR22] FAO/WHO (2012) Report of the 6th session of the Codex Committee of food additives and contaminants-proposed draft maximum levels for arsenic in rice. Joint FAO/WHO food standards programme codex alimentarius commission, 35th Session, Rome, Italy, 2–7 July 2012

[CR23] Ferri T, Favero G, Frasconi M (2007). Selenium speciation in foods: preliminary results on potatoes. Microchem J.

[CR24] Fordyce FM, Johnson CC, Navaratna URB, Appleton JD, Dissanayake CB (2000). Selenium and iodine in soil, rice and drinking water in relation to endemic goitre in Sri Lanka. Sci Total Environ.

[CR25] Fu J, Zhou Q, Liu J, Liu W, Wang T, Zhang Q, Jiang G (2008). High levels of heavy metals in rice (*Oryza sativa* L.) from a typical E-waste recycling area in southeast China and its potential risk to human health. Chemosphere.

[CR26] Gilbert PJ, Polya DA, Cooke DA (2015). Arsenic hazard in Cambodian rice from a market-based survey with a case study of Preak Russey village, Kandal Province. Environ Geochem Health.

[CR27] Gunaratne A, Wu K, Li D, Bentota A, Corke H, Cai Y-Z (2013). Antioxidant activity and nutritional quality of traditional red-grained rice varieties containing proanthocyanidins. Food Chem.

[CR28] Gupta UC, Gupta SC (1998). Trace element toxicity relationships to crop production and livestock and human health: implications for management. Commun Soil Sci Plant Anal.

[CR29] Haug A, Graham RD, Christophersen OA, Lyons GH (2007). How to use the world’s scarce selenium resources efficiently to increase the selenium concentration in food. Microb Ecol Health Dis.

[CR30] Ja Tan, Zhu W, Wang W, Li R, Hou S, Wang D, Yang L (2002). Selenium in soil and endemic diseases in China. Sci Total Environ.

[CR31] Jayasumana MACS, Paranagama PA, Amarasinghe MD, Wijewardane KMRC, Dahanayake KS, Fonseka SI, Rajakaruna KDLMP, Mahamithawa AMP, Samarasinghe UD, Senanayake VK (2013). Possible link of chronic arsenic toxicity with chronic kidney disease of unknown etiology in Sri Lanka. J Nat Sci Res.

[CR32] Jayasumana C, Paranagama P, Fonseka S, Amarasinghe M, Gunatilake S, Siribaddana S (2015). Presence of arsenic in Sri Lankan rice. Int J Food Contam.

[CR33] Jayatilake N, Mendis S, Maheepala P, Mehta FR (2013). Chronic kidney disease of uncertain aetiology: prevalence and causative factors in a developing country. BMC Nephrol.

[CR34] Kelly S, Baxter M, Chapman S, Rhodes C, Dennis J, Brereton P (2002). The application of isotopic and elemental analysis to determine the geographical origin of premium long grain rice. Eur Food Res Technol.

[CR35] Kennedy G, Burlingame B (2003). Analysis of food composition data on rice from a plant genetic resources perspective. Food Chem.

[CR36] Khaniki GR, Zozali MA (2005). Cadmium and lead contents in rice (Oryza sativa) in the North of Iran. Int J Agric Biol.

[CR37] Li R, Stroud J, Ma J, Mcgrath S, Zhao F (2009). Mitigation of arsenic accumulation in rice with water management and silicon fertilization. Environ Sci Technol.

[CR38] Lyons GH, Judson GJ, Ortiz-Monasterio I, Genc Y, Stangoulis JC, Graham RD (2005). Selenium in Australia: selenium status and biofortification of wheat for better health. J Trace Elem Med Biol.

[CR39] Mahatantila K, Chandrajith R, Jayasena HAH, Ranawana KB (2008). Spatial and temporal changes of hydrogeochemistry in ancient tank cascade systems in Sri Lanka: evidence for a constructed wetland. Water Environ J.

[CR40] Meharg AA, Rahman MM (2003). Arsenic contamination of Bangladesh paddy field soils: implications for rice contribution to arsenic consumption. Environ Sci Technol.

[CR41] Meharg AA, Williams PN, Adomako E, Lawgali YY, Deacon C, Villada A, Cambell RCJ, Sun G, Zhu Y-G, Feldmann J (2009). Geographical variation in total and inorganic arsenic content of polished (white) rice. Environ Sci Technol.

[CR42] Meharg AA, Norton G, Deacon C, Williams P, Adomako EE, Price A, Zhu Y, Li G, Zhao FJ, McGrath S (2013). Variation in rice cadmium related to human exposure. Environ Sci Technol.

[CR43] Muthayya S, Sugimoto JD, Montgomery S, Maberly GF (2014). An overview of global rice production, supply, trade, and consumption. Ann N Y Acad Sci.

[CR44] Nanayakkara S, Komiya T, Ratnatunga N, Senevirathna STMLD, Harada KH, Hitomi T, Gobe G, Muso E, Abeysekera T, Koizumi A (2012). Tubulointerstitial damage as the major pathological lesion in endemic chronic kidney disease among farmers in North Central Province of Sri Lanka. Environ Health Prev Med.

[CR45] Nanayakkara S, Senevirathna STMLD, Abeysekera T, Chandrajith R, Ratnatunga N, Gunarathne EDL, Yan J, Hitomi T, Muso E, Komiya T (2014). An integrative study of the genetic, social and environmental determinants of chronic kidney disease characterized by tubulointerstitial damages in the North Central Region of Sri Lanka. J Occup Health.

[CR46] Neal RH, Sposito G (1989). Selenate adsorption on alluvial soils. Soil Sci Soc Am J.

[CR47] Norton GJ, Islam MR, Duan G, Lei M, Zhu Y, Deacon CM, Moran AC, Islam S, Zhao FJ, Stroud JL (2010). Arsenic shoot-grain relationships in field grown rice cultivars. Environ Sci Technol.

[CR48] Panabokke CR, Sakthivadivel R, Weerasinghe AD (2002). Small tanks in Sri Lanka: evolution, present status, and issues.

[CR49] Paroda R (1999). Genetic diversity, productivity, and sustainable rice production. 19th Session of the International Rice Commission.

[CR50] Rayman MP (2000). The importance of selenium to human health. Lancet.

[CR51] Sun HJ, Rathinasabapathi B, Wu B, Luo J, Pu LP, Ma LQ (2014). Arsenic and selenium toxicity and their interactive effects in humans. Environ Int.

[CR52] Takahashi Y, Minamikawa R, Hattori KH, Kurishima K, Kihou N, Yuita K (2004). Arsenic behavior in paddy fields during the cycle of flooded and non-flooded periods. Environ Sci Technol.

[CR53] Todorov V, Filzmoser P (2010). Robust statistic for the one-way MANOVA. Comput Stat Data Anal.

[CR54] Underwood EJ (1979). Trace elements and health: an overview. Philos Trans R Soc Lond B Biol Sci.

[CR55] Watanabe T, Shimbo S, Moon CS, Zhang ZW, Ikeda M (1996). Cadmium contents in rice samples from various areas in the world. Sci Total Environ.

[CR56] White PJ, Broadley MR (2009). Biofortification of crops with seven mineral elements often lacking in human diets–iron, zinc, copper, calcium, magnesium, selenium and iodine. New Phytol.

[CR57] WHO, FAO (2002). Diet, nutrition and the prevention of chronic diseases.

[CR58] Williams PN, Villada A, Deacon C, Raab A, Figuerola J, Green AJ, Feldmann J, Meharg AA (2007). Greatly enhanced arsenic shoot assimilation in rice leads to elevated grain levels compared to wheat and barley. Environ Sci Technol.

[CR59] Williams PN, Lombi E, Sun GX, Scheckel K, Zhu YG, Feng X, Zhu J, Carey AM, Adomako E, Lawgali Y (2009). Selenium characterization in the global rice supply chain. Environ Sci Technol.

[CR60] WHO (1996). Trace elements in human nutrition and health.

[CR61] Yang X, Ye ZQ, Shi CH, Zhu ML, Graham RD (1998). Genotypic differences in concentrations of iron, manganese, copper, and zinc in polished rice grains. J Plant Nutr.

[CR62] Zhang ZW, Moon CS, Watanabe T, Shimbo S, Ikeda M (1998). Contents of nutrient and pollutant elements in rice and wheat grown on the neiboring frelds. Sci Total Environ.

[CR63] Zhu YG, Williams PN, Meharg AA (2008). Exposure to inorganic arsenic from rice: a global health issue?. Environ Pollut.

